# Effect of dental restorations and prostheses on radiotherapy dose distribution: a Monte Carlo study

**DOI:** 10.1120/jacmp.v10i1.2853

**Published:** 2009-02-03

**Authors:** David W. H. Chin, Nathaniel Treister, Bernard Friedland, Robert A. Cormack, Roy B. Tishler, G. Mike Makrigiorgos, Laurence E. Court

**Affiliations:** ^1^ Department of Radiation Oncology Dana‐Farber/Brigham and Women's Cancer Center and Harvard Medical School Boston MA U.S.A.; ^2^ Dana‐Farber/Brigham and Women's Cancer Center and Harvard School of Dental Medicine Boston MA U.S.A.; ^3^ Harvard School of Dental Medicine Boston MA U.S.A.

**Keywords:** Monte Carlo, radiotherapy, backscatter, dental work, mucositis

## Abstract

Dental restorations, fixed prosthodontics, and implants affect dose distribution in head and neck radiation therapy due to the high atomic number of the materials utilized. The backscatter of electrons from metallic materials due to the impinging treatment x‐ray results in localized dose enhancements. These dose enhancements cause localized mucositis in patients who have dental work, a significant clinical complication. We investigated the backscatter effect of 23 configurations of dental work using the EGS4nrc Monte Carlo (MC) simulation system. We found that all‐metal fixed partial dentures caused the highest amount of dose enhancement – up to 33% – while amalgam restorations did not cause a significant amount. Restorations with a ceramic veneer caused up to 8% enhancement. Between 3 mm and 5 mm of water‐equivalent material almost completely absorbed the backscatter. MC simulations provide an accurate estimate of backscatter dose, and may provide patient‐specific estimates in future.

PACS: 87.10.Rt, 87.53.Bn

## I. INTRODUCTION

In radiotherapy of the head and neck, mucositis is frequently observed adjacent to metallic dental restorations. The therapy beam scatters electrons from the high‐Z metals used in dental alloys, resulting in a local dose enhancement. The effect is local due to the short range of the scattered electrons in tissue. This local dose enhancement leads to excess dose in the buccal mucosa causing mucositis. The backscatter dose enhancement and its prevention are of clinical interest and have been extensively studied.^(^ ^1^ ^–^ ^12^ ^)^

Here, we focus on those restorations which may come into contact with the oral mucosa. Farahani et al.^(1)^ performed experiments to measure the effects of various dental restoration materials on dose distribution: 18‐karat gold alloy, Ag‐Hg amalgam, Ni‐Cr alloy, human tooth tissue, and soft‐tissue‐simulating polymer. We chose to concentrate on these experiments because they performed measurements in geometries which simulated a human dental arch, rather than isolated pieces of material in geometric phantoms. (See Table 1) Their dosimetry was obtained using GafChromic (International Specialty Products, Wayne, NJ, U.S.A.) film. The two experiments consisted of three setups:

**Table 1:** List of materials and their corresponding PEGS data name. The PEGS data for dental amalgam, Eclipse alloy, and Ceramco C3 ceramic veneer were generated based on manufacturers' data sheets and the XCOM photon cross section data tables.^(21)^ The densities of the 700ICRU materials were obtained from BEAM4nrc.^(16)^

*Material*	*PEGS data*	*Density* $\left(g/{\text{cm}}^{3}\right)$
Tissue	ICRUTISSUE700ICRU	1.00
Bone/tooth	ICRPBONE700ICRU	1.101 – 2.088
Amalgam	DENTALAMALGAM	8.0
Eclipse alloy	ECLIPSE	13.8
Ceramco C3	CERAMCOC3	2.6
Steel	STEEL700ICRU	8.1
Air	AIR700ICRU	0.001 – 0.044

(a) 2 mm thick samples of the materials mentioned above were placed between two stacks of GafChromic film, to distances of about $500\;\text{mg}/{\text{cm}}^{2}$ on the backscatter and forward‐scatter sides (Note: To convert $\text{mg}/{\text{cm}}^{2}$ to equivalent distance in material, divide by the density of the material.)
(b) three human teeth were embedded in epoxy to simulate a dental arch; they were arranged in a row: the first with no restoration, the second with a gold alloy crown, and the third with an amalgam restoration
(c) as in (b), but the second tooth had a Ni‐Cr crown, instead of gold alloy.

These phantoms were irradiated with Co60 and 10 MV photon beams.

Dose enhancement was found to be as great as 100% on the backscatter side of the gold alloy crown. In comparison, the unrestored tooth enhanced dose by 20%. This dose enhancement decayed to less than 5% at a distance of about $300\;\text{mg}/{\text{cm}}^{2}$ (3 mm in material of density $1\;g/{\text{cm}}^{3}$).

In the present study, we performed a Monte Carlo (MC) dosimetry study to understand the effects of various forms of dental work on dose distribution, and to gauge the efficacy of different forms of shielding. The EGS4nrc system^(^ ^13^ ^–^ ^15^ ^)^ was used to simulate a 6 MV therapy beam, typical for head and neck radiotherapy, and to calculate the dose distribution in different virtual phantoms. We used 21 different combinations of materials and geometry in the phantoms, and 2 patient scans. The advantage of using a MC simulation over an experiment is that we were able to investigate many materials and geometries while avoiding painstaking measurements. Furthermore, new materials may be investigated in the future using the same experimental parameters, and patient‐specific MC simulation may also be performed to discover if there is a need for preventative action.

## II. MATERIALS AND METHODS

### A. Monte Carlo software

The EGS4nrc MC code BEAMnrc^(16)^ was used to simulate a 6MV x‐ray beam from a Varian 21EX linac. The beam size was set to be 10 cm×10 cm at isocenter, and SSD was 97.00 cm. Dose deposition was simulated with the DOSXYZnrc^(17)^ code.

### B. Validation

The EGS4nrc codes BEAMnrc and DOSXYZnrc have been validated for open fields and intensity‐modulated fields, including a 7‐field IMRT plan simulated on CT data sets of a cylindrical phantom and a RANDO (The Phantom Laboratory, Salem, NY) anthropomorphic phantom. The validations compared MC results with measurements using ion chambers and thermoluminescent dosimeters (TLDs), giving discrepancies of less than 2%.^(18)^

**Figure 1:**
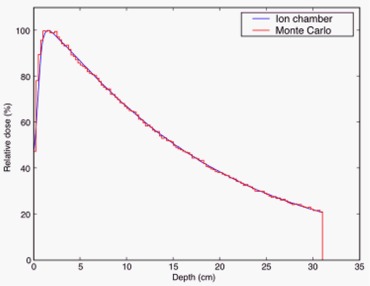
Commissioning PDD measurement using ion chamber and Monte Carlo simulation. The measurements were taken at 1 mm intervals, while the simulation data had a resolution of 2.5 mm. The maximum estimate on statistical error for the simulation was 1.5%.

Our installation of EGS4nrc was previously commissioned and shown to agree with experiment within 2% for open beam, and within 3% for intensity‐modulated radiation therapy (IMRT) using ionization chambers and TLDs.^(19)^ Part of this validation compared MC simulations with ion chamber measurements taken during commissioning. These were measurements of relative dose profiles and percent depth dose (PDD) for various field sizes in a Wellhöfer (IBA Dosimetry America, Bartlett, TN, U.S.A.) 48cm×48cm×48cm water tank. The PDD measurement data and the MC simulation results are plotted in Fig. 1. The MC simulation had a maximum estimated statistical error of 1.5%, and agreed well with the measurement.

**Figure 2:**
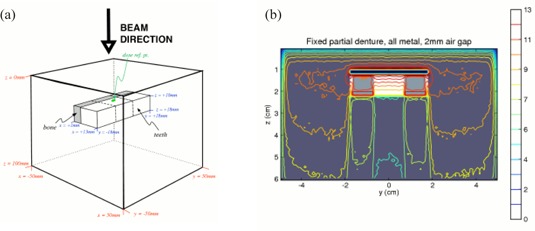
Schematic of phantom configuration. The material compositions used were: ICRUTISSUE700ICRU with density $1\;g/{\text{cm}}^{3}$ for tissue, and ICRPBONE700ICRU with density $1.6\;g/{\text{cm}}^{3}$ for both bone and teeth. The distance between the teeth and the top surface of the phantom is 10 mm. The coordinates of the dose reference point are (5.5 mm, 5.5 mm, 9.5 mm). The thickness of the bone layer is 4 mm in the x‐direction. The lower an x‐z cross‐section (y=5.5mm) of the 3D dose distribution for the fixed partial denture phantom, with 2 mm air gap. The dose scale is in arbitrary units.

A total of $1.1\times 10^{9}$ particles were used, resulting in a phase space after the linac head containing $5.8\times 10^{7}$ particles. This phase space file was then used in the DOSXYZnrc simulation to calculated deposited dose in the phantoms described below. See Fig. 2 for a representative example.

### C. Dental restoration materials

There is a wide variety of materials used in dental restorations and prostheses. We chose commonly used materials that included:

(a) dental amalgam: a mixture of silver (69.3%), tin (17.9%), copper (11.8%), and zinc (1.0%)
(b) Eclipse (Dentsply Ceramco, Inc., York, PA, U.S.A.) alloy: an alloy of gold (52.0%), palladium (37.5%), zinc (4.0%), indium (3.0%), tin (3.0%), and rhenium (0.5%)
(c) Ceramco C3 ceramic enamel (Dentsply Ceramco, Inc., York, PA, U.S.A.): sodium potassium aluminosilicate ${\text{Na}}_{2}K[{\text{Al}}_{3}{\text{Si}}_{3}O_{12}]$ (the MSDS for Ceramco C3 lists 80% – 100% of this material, and $0\%-20\%\;{\text{SnO}}_{2})$

Dental amalgam is used in the restoration of teeth following caries removal, and in some cases may replace greater than 50% of the coronal structure. Eclipse alloy is used in full coverage crowns, where the restored tooth is clad in the alloy. Ceramic restorations are used for cosmetic reasons as they closely simulate the appearance of dental enamel. These are most commonly fused to an alloy substrate, although full ceramic restorations are available (for example, in the case of cosmetic veneers).

A total of 21 geometrical phantom configurations were studied, and were divided into 3 groups based on the amount of air gap inserted between the teeth and the tissue. The air gaps served to attenuate the backscatter dose to the mucosa:

(a) no air gap
(b) 2 mm air gap
(c) 5 mm air gap

Ignoring the varying air gaps, the 7 phantom configurations were:

(a) no dental work
(b) edentulous (i.e. tooth removed and replaced with tissue‐equivalent material)
(c) amalgam restoration
(d) amalgam restoration with an exposed amalgam surface on the buccal (beam‐side) aspect
(e) fixed partial denture (FPD) with ceramic veneer
(f) all‐metal FPD
(g) gold alloy crown

In addition, 2 simulations were performed on patient CT data:

(a) raw patient CT data
(b) patient CT data with simulated gold alloy crown inserted.

## D. Phantom configurations

We manually created EGSPHANT‐format^(17)^ simulated CT phantoms with different configurations and materials. This allowed us the flexibility of defining various geometries and materials as needed. The phantoms consisted of a 10cm×10cm×10cm block of simulated tissue, with density $1\;g/{\text{cm}}^{3}$. The simulated teeth were cuboids of dimensions 12mm×8mm×8mm. This size was used based on approximate measurements of this author's teeth. A layer of simulated bone (4 mm thick) was placed on the inferior side of the row of simulated teeth. Since the voxel size was set to be 1mm×1mm×1mm, the minimum thickness of any layer was 1 mm. See Fig. 2.

**Figure 3:**
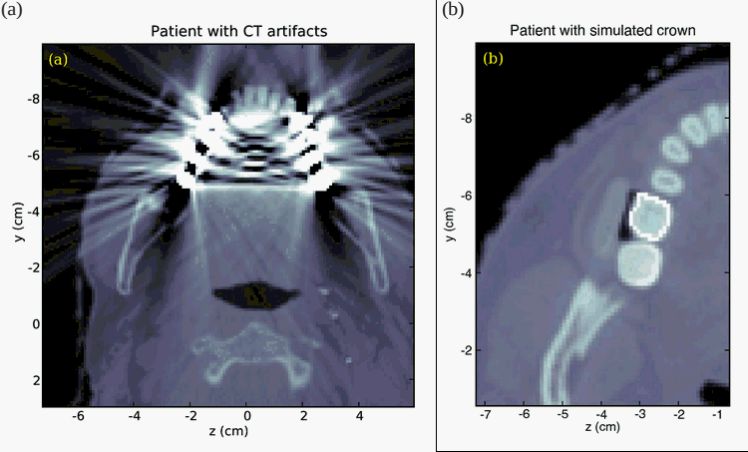
CT scan slices of two patients. The first patient scan (a) showed artifacts due to dental work. The second patient (b) had no dental work; a simulated gold alloy crown was inserted into the CT volume.

For the two simulations on patient CT scans, one was done with a simulated gold crown inserted into the CT data Fig. 3(a), and one without. Fig. 3(b) The simulated crown was inserted by manipulating the material and density data in the EGSPHANT file. We did not perform a simulation on a patient with actual dental work due to the significant amount of artifacts in the CT scan. (See Fig. 3a) In such a case, the typical clinical approach would be to contour the artifacts, and to set the contoured volume to water. While this would have removed the artifacts and allowed for a clean MC simulation, it would also have removed all anatomical structure and heterogeneity, reducing the problem to the geometrical phantoms described above. Even if the artifacts were not contoured out, structural details of the dental work would not be visible in the CT.

## III. RESULTS AND DISCUSSION

**Figure 4:**
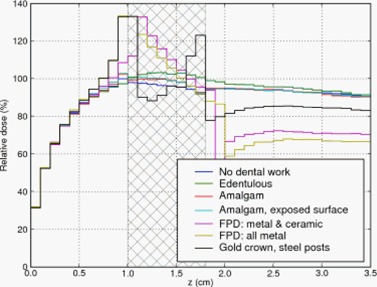
Depth dose curve: no air gap between teeth and tissue. Cross‐hatched region represents teeth.

**Figure 5:**
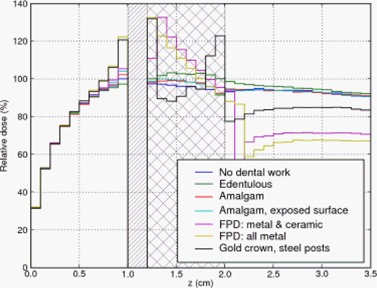
Depth dose curve: 2 mm air gap between teeth and tissue. Diagonally hatched region represents air gap.

**Figure 6:**
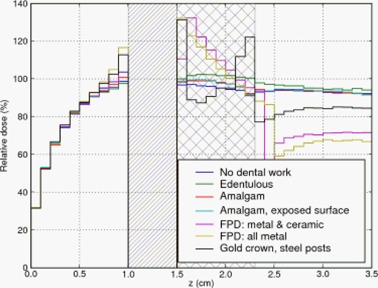
Depth dose curve: 5 mm air gap between teeth and tissue.

Figures 4–6 show the relative depth‐dose curves along a line passing through the center of the middle simulated tooth. In all three plots, the doses are expressed relative to the dose in the tissue immediately adjacent to the middle tooth (i.e. at coordinates 5.5 mm, 5.5 mm, 9.5 mm) for the case with no dental work and no air gap.

The cross‐hatched region in the three Figures represent the extent of the teeth. For the edentulous case (i.e. no tooth or prosthesis present) we filled the gap with tissue‐equivalent material. The diagonally hatched region in Figs. 5 and 6 represent an air gap interposed between the buccal mucosa and the teeth. Dose in the air gap was set to zero via a standard option in DOSXYZnrc.

In the first case, Fig. 4, the fixed partial denture (FPD) with an all metal pontic (bridging prosthetic tooth) and the gold alloy crown caused the largest dose enhancement of 33% and 32% (respectively) relative to the case without dental work. The FPD with a ceramic veneer over the pontic resulted in a lower dose enhancement of 8%.

The amalgam restoration with exposed amalgam on the buccal aspect did not produce much backscatter; the dose adjacent to the tooth was within 2% of that in the case of the unrestored tooth and the amalgam restoration without an exposed buccal surface. The edentulous case showed slightly lower dose than the unrestored tooth, as expected, because tissue‐equivalent material has a lower average Z than teeth.

In Fig. 5, a 2 mm air gap is interposed between the teeth and the mucosa. The FPD with all‐metal pontic and gold alloy crown again resulted in the largest dose enhancement – 22% and 20% respectively. The FPD with ceramic veneer again had an intermediate amount of dose enhancement, and the remaining materials produced little enhancement.

The final set of MC data with geometrical phantoms is plotted in Fig. 6. Here, the FPD with all‐metal pontic gave an enhancement of 16%. The enhancement due to the gold crown was 12%. The remaining materials gave no more than 5% enhancement.

Since the simulations showed that the short‐range backscatter enhancement were significantly reduced by air, which has low density, we examined the PDD as a function of distance from the tooth in the direction of decreasing z (i.e. up‐beam). We expected the PDD here in the tissue‐equivalent water‐density material to be a sum of the photon build‐up and the electron backscatter enhancement. That is, the relative dose should fall quicker than it did in the air gap.

The variation of the PDD with the distance from the tooth, and with varying amounts of air gap, is shown in Table 2. In all cases, we see a reduction in dose as the distance increases. This is consistent with the monotonic nature of the buildup PDD plus backscatter dose.

**Table 2:** Relative dose (%) for various configurations of materials and geometry, with varying amounts of air gap between the simulated teeth and the buccal mucosa. Reference dose is taken to be the dose 0.5 mm from the tooth (up‐beam direction) in the “No dental work” configuration. The values in this table are the average of doses in six voxels adjacent to the tooth on the buccal aspect (i.e. z=0.95,y=[−0.25,0.25],x=0.55).

*Distance from tooth*	*0.5 mm up‐beam*			*2.5 mm up‐beam*			*3.5 mm up‐beam*		
Air gap (mm)	0	2	5	0	2	5	0	2	5
No dental work	100	100	98	93	93	93	90	90	90
Edentulous	97	97	98	93	94	93	90	90	90
Amalgam	102	102	100	93	94	95	90	90	91
Amalgam, exp. surf.	102	104	103	95	95	94	92	91	90
FPD	108	105	103	95	95	95	91	91	91
FPD, all metal	133	122	116	100	99	98	94	94	93
Crown	132	120	112	100	99	97	94	93	92

In the cases with little or no backscatter enhancement, the dose is reduced by about 10% at a distance of 3.5 mm. In the cases that do show backscatter enhancement, the reductions in dose at 3.5 mm are much greater. For gold and the FPD with all‐metal pontic, the dose is reduced by 29% to 94%, just 4% higher than the dose in the case without dental work.

When an air gap of 2 mm is introduced, the dose reduction behavior is similar. The relative doses at 3.5 mm up‐beam for the various configurations are almost identical to the case where there was no air gap. This is true for the cases with a 5 mm air gap, as well.

**Figure 7:**
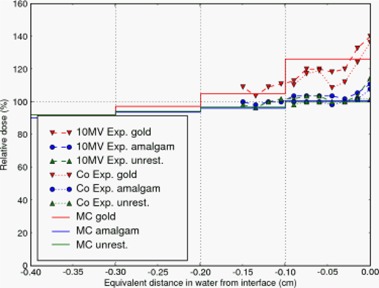
A plot of the relative doses as measured by Farahani et al.^(1)^ where the teeth were embedded in epoxy to simulate a dental arch. Doses calculated by Monte Carlo are also shown.

We compared results of the simulation with the experiment in Farahani et al.^(1)^ which modeled a dental arch, as described in Sec. I. The phantoms were irradiated with Co60 and 10 MV photon beams. These results, with appropriate distance scaling, are plotted together with results from the MC simulations in Fig. 7. The spatial resolution of the simulations was necessarily coarser to limit the statistical uncertainty in computed dose per voxel while retaining a reasonable computation time. In addition, the simulation used a 6 MV beam corresponding to clinical procedure at this institution. Given the differences between the experiment and the MC simulation, we found a reasonable correspondence between the simulations and the experiment.

The Monte Carlo simulations also reproduced the short‐range nature of the backscatter dose enhancement. In Table 2, all 21 combinations of geometry and air gap showed a reduction in the relative dose at a distance of 3.5 mm up‐beam to the tooth‐mucosa interface. In those cases with a large amount of backscatter (gold, and FPD with all‐metal pontic), the doses decreased such that they were just 4% higher than the dose in the case without dental work. We infer that the backscatter effect has almost completely disappeared 3.5 mm into the mucosa. This is consistent with a dose increment due to scattered secondary electrons.^(1)^

Since backscatter dose is due to scattered electrons, we expect to observe effective shielding even with low‐Z materials. Indeed, the MC simulations display this effect. In Fig. 4, the FPD with a ceramic veneer over the pontic resulted in a dose enhancement of only 8% compared to 32% caused by the gold crown, and 33% by the FPD with an all‐metal pontic. That is, the 1 mm ceramic veneer provided shielding from the backscattered electrons. As low‐energy electrons have limited range in air, we expect some reduction in backscatter if an air gap is interposed between the tooth and the mucosa. Figs. 5 and 6 and Table 2 confirm this expectation: a 2 mm air gap reduced the dose enhancement of the worst cases by a third, and a 5 mm air gap caused greater reduction.

The MC simulation results indicate that dental work with exposed gold alloys are of most clinical concern: all‐metal FPDs and gold alloy crowns cause the largest amount of dose enhancement. Amalgam restorations, on the other hand, do not cause large backscatter dose enhancements and hence are not of clinical concern. For those cases of concern, it is the area of exposed metal surface which causes backscatter (i.e. it is a surface effect). The amount of metal below the surface dose, being self‐shielded, does not contribute to backscatter.

**Figure 8:**
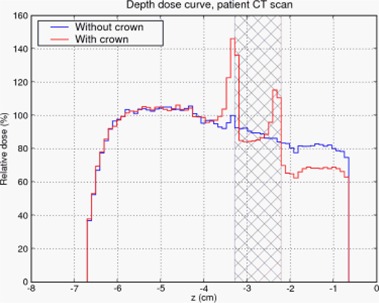
Depth dose curves of patient without dental work and patient with simulated gold alloy crown. Cross‐hatched region represents extent of the tooth. (See Fig. 3b)

In order to understand the effect of backscatter in patients, we performed two simulations. Using the CT scan of a patient without dental work, we created another phantom with dental work by manipulating the CT data, inserting a simulated gold alloy crown. (See Fig. 3b). Identical treatments were then simulated to both phantoms, resulting in the dose profiles in Fig. 8. The backscatter enhancement due to the crown was >40%, and the enhancement decayed completely within 5 mm in the up‐beam direction. These results are consistent with the idealized geometrical phantom simulations above.

## IV. CONCLUSIONS

The occurrence of mucositis in treatment of head and neck cancers is a significant clinical issue. There have been experimental studies which quantified the backscatter dose enhancement which leads to mucositis, and which explored methods of reducing this effect.^(1)^ ^,^ ^(2)^ ^,^ ^(20)^ However, due to the difficulty in making the dose measurements, only a small number of configurations were studied.

We have studied 21 geometrical configurations of simulated teeth and dental work to quantify the effects of backscatter. We also studied the effects of backscatter in a patient by inserting a simulated gold alloy crown and comparing the results to the unmodified patient simulation. The amount of dose enhancement and the distance it penetrated were consistent with the dose characteristics in the geometric phantoms above. We found that 3 mm of water‐density (low‐Z) material would adequately shield the oral mucosa from excess dose. This is consistent with the recommendations of Farahani et al.^(1)^ ^,^ ^(2)^ and Reitemeier et al.^(20)^ to use a low‐Z shield of thickness approximately equal to $0.3\;g/{\text{cm}}^{2}$.

Our results also indicate that a specialized shield is not necessary. The attenuation of the backscatter dose by the air gap shows that even materials of low density would be sufficient. This is further reinforced by the attenuation within the buccal mucosa: the backscatter dose is completely attenuated within about 4 mm. Thus, a cotton roll which has been soaked in water will be sufficient to shield the mucosa.

Monte Carlo simulations provide an accurate way of estimating the backscatter dose enhancement. Simulations also allow different configurations of teeth and dental work to be easily studied compared to experiment. Patient‐specific simulations are also possible.

## Supporting information

Supplementary Material
