# Supplementary figures and images for: Effect of dental restorations and prostheses on radiotherapy dose distribution: a Monte Carlo study
Source: J Appl Clin Med Phys. 2009 Feb 3;10(1):80–9. doi: 10.1120/jacmp.v10i1.2853 (PMC5720502; doi:10.1120/jacmp.v10i1.2853)

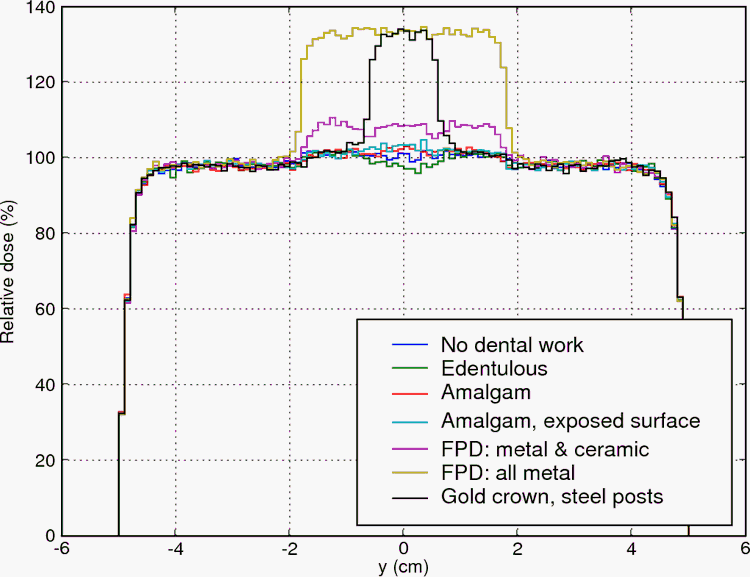

Supplement: Supplementary file 1 — Supplementary Material [file ACM2-10-080-s001.gif]
